# Data-Driven Material Selection for Flexible Wearable Sensors Under Environmental Coupling Conditions

**DOI:** 10.3390/s26072122

**Published:** 2026-03-29

**Authors:** Yanping Lu, Myun Kim, Hanwen Zhang

**Affiliations:** Department of Industrial Design, Pukyong National University, 45, Yongso-ro, Nam-Gu, Busan 48513, Republic of Korea; luyanping@pukyong.ac.kr (Y.L.); zhanghanwen@pukyong.ac.kr (H.Z.)

**Keywords:** environmental coupling, flexible electronic wearable materials, green clothing, sensors, material selection recommendation model

## Abstract

Flexible wearable electronics have shown strong potential for medical and health monitoring; however, conventional materials often fail to simultaneously satisfy the requirements of signal stability, wear comfort, and environmental adaptability under dynamic use conditions. To address this issue, this study proposes a data-driven material selection framework for flexible wearable sensors based on the extreme gradient boosting (XGBoost) algorithm. The model integrates user perception, material physical parameters, and environmental coupling performance indicators to enable intelligent material matching and recommendation. Experimental results show that the proposed model achieves a recommendation accuracy of 94.5%, outperforming conventional comparison methods. Among the candidate materials, silver nanowires (AgNWs) exhibit superior overall performance, including a higher signal-to-noise ratio, lower skin-contact impedance, and stronger sweat resistance. In physiological monitoring experiments, the maximum deviation of the sensor response was below 3% under both static and motion conditions. In environmental coupling tests, the recommended material improved the system signal-to-noise ratio by 68% and reduced 24-h sensitivity decay by 75%. These results indicate that the proposed XGBoost-based framework can effectively support material selection for flexible wearable sensors and improve signal reliability and environmental adaptability in complex application scenarios.

## 1. Introduction

Flexible electronic wearables have shown great application promise in domains including sports science, personalized health management, and healthcare monitoring, driven by the rapid development of smart wearable technologies [1]. However, existing wearable devices still face numerous challenges in practical applications. Medical monitoring scenarios demand higher standards for the accuracy, reliability, and long-term wearing comfort of wearable devices, yet traditional materials often struggle to meet these requirements simultaneously. For example, factors such as motion artifacts, sweat-induced corrosion, and mechanical stretching can all lead to signal distortion or device failure, thereby affecting the accuracy of monitoring data [2]. Consequently, the optimization of the performance of flexible electronic wearable materials through intelligent material selection methods as well as the achievement of effective coupling with the human body and the environment have become pivotal issues in current research. Recent years have witnessed a marked advancement in the realm of research concerning flexible electronic materials. The advent of novel materials, such as graphene, silver nanowires (AgNWs), and conductive polymers, has precipitated considerable interest due to their superior conductivity, flexibility, and biocompatibility. However, the performance advantages of a single material are insufficient to meet the comprehensive requirements of complex application scenarios [3,4]. Thus, a rigorous and effective material selection approach that can thoroughly assess material performance characteristics and facilitate tailored recommendations is urgently needed. The extreme gradient boosting (XGBoost) algorithm has exhibited considerable advantages in the domain of recommendation systems, attributable to its advanced feature selection capability and its high-precision prediction performance.

In their study, Song et al. proposed a method to enhance the conductivity of polymer films using dopants and applied them to methyl phosphonate sensors. The results showed that this sensor showed low power consumption and alarm capabilities, making it suitable for use in wearable devices. Moreover, PQTS12 doped with FeCl_3_ (PQTS12) exhibited the highest doping efficiency, with a significant increase in conductivity. After 3000 bending cycles, the conductivity degradation was only 9.2% [5]. The use of different materials in device fabrication can significantly enhance the application performance of the devices. However, the environmental coupling effects of these materials still require further investigation. To better understand the evolution of flexible piezoelectric composite materials and to propel the creation of next-generation flexible electronic devices, Zhang J et al. established a research framework in their study. According to their findings, flexible piezoelectric composite materials offer special benefits including high consistency and may find application as essential components in piezoelectric sensors. These materials have been applied in multiple fields, such as underwater detection. These studies have completely advanced traditional piezoelectric material design concepts and application scenarios. Additionally, this study anticipated and reviewed current problems and potential future developments of these materials [6]. Although flexible electronic wearable materials have been widely investigated, material selection for practical sensing applications remains a challenging problem [7]. In real-world wearable scenarios, candidate materials must be evaluated not only in terms of conductivity or flexibility but also considering multiple coupled factors such as skin–electrode impedance, signal stability under motion, sweat resistance, conformability, and long-term wearing comfort [8,9]. This makes material selection a typical multicriteria decision-making problem. Therefore, an intelligent, data-driven method is needed to rank candidate materials by jointly considering user requirements, physical material properties, and environmental coupling performance [10]. XGBoost is well suited to this task because it can efficiently model nonlinear relationships among heterogeneous tabular features, provide robust predictive performance, and offer feature-importance information that supports interpretable recommendations [11]. Based on these advantages, this study introduces XGBoost as the core engine for material screening and recommendation in flexible wearable sensing applications.

Accordingly, this study analyzes the environmental coupling performance of flexible electronic wearable materials and proposes an XGBoost-based material selection framework for intelligent recommendation. By integrating user perception, material physical parameters, and environmental coupling indicators, the proposed model enables data-driven screening of candidate materials for wearable sensing applications. The main contributions of this study are threefold. First, a multidimensional evaluation system is constructed to jointly characterize user requirements, material properties, and environmental coupling performance. Second, an XGBoost-based recommendation framework is developed for intelligent material matching and ranking. Third, a composite fabric electrode structure is designed and experimentally validated for physiological monitoring under static and motion conditions.

## 2. Methods and Materials

### 2.1. Construction of a Material Selection Model Based on Clothing Perception

**Figure 1:**
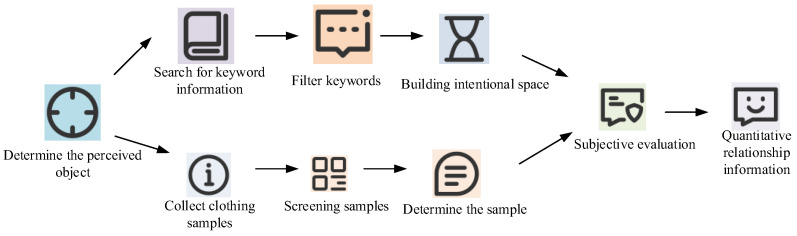
Perception evaluation process (Module source in the figure: https://iconpark.oceanengine.com/home, accessed on 8 January 2026).

This study focuses on smart medical wearable clothing as the research object and establishes a clothing material perception-based selection model. First, the materials of the clothing are evaluated and analyzed based on the different user groups. The evaluation method primarily relies on the description and analysis of users’ perceptual intentions. In perceptual evaluations, users typically use adjectives such as “beautiful” and “elegant” to evaluate and perceive clothing. These terms are used to construct an intention space [12,13]. Figure 1 shows the perceptual evaluation process.

In Figure 1, during the perceptual evaluation stage, an initial intention space is first constructed based on users’ subjective descriptions of wearable clothing materials. To improve semantic coverage, we established a multidimensional material perception lexicon containing both general perceptual descriptors and textile structure-related terms, such as softness, breathability, elasticity, durability, and skin conformity. User inputs collected through structured questionnaires are standardized and mapped to the corresponding material attribute space. These perceptual features are then integrated with the analytic hierarchy process and the XGBoost model to achieve structured matching between user intentions and material parameters. A total of 32 participants were enrolled in the perceptual questionnaire survey, and responses were quantified using a 5-point Likert scale.

The XGBoost algorithm is capable of processing and analyzing data with medium to low dimensionality. It learns from the evaluation data of all clothing samples and applies a learned model to analyze the results. Equation (1) represents the learning model calculation formula for the algorithm [14,15].

(1) $$y_{i}=\sum_{k=1}^{K}f_{k}(x_{i})$$

In Equation (1), $y_{i}$ is the final PV of the i-th sample. K is the total number of base learners. $f_{k}(x_{i})$ is the PV of the i-th sample of the K-th base learner. $x_{i}$ represents the input features of the i-th sample. The construction process of the algorithm model consists of four steps. First, the objective function is determined, as shown in Equation (2) [16,17].

(2) $$Obj=\sum_{I=1}^{n}L({\ddot{y}}_{i},y_{i})+\sum_{k=1}^{K}\upsilon (f_{k})$$

In Equation (2), $L({\ddot{y}}_{i},y_{i})$ is the loss function (LF). ${\ddot{y}}_{i}$ is the true value. $\upsilon (f_{k})$ represents the regularization term, which prevents the model from overfitting.

Equation (3) displays the expression for the regularization term.

(3) $$\upsilon (f_{k})=\lambda T+\frac{1}{2}\varphi {\left\|w\right\|}^{2}$$

In Equation (3), λ represents the minimum loss reduction required for a leaf split. T is the number of leaf nodes (LNs) in the tree. φ represents the regularization coefficient. w is the weight of the LN. The model objective function is expanded using the Taylor formula to obtain Equation (4) [18,19].

(4) $$Obj^{\left(t\right)}\approx \sum_{I=1}^{n}\left[L{\left({\ddot{y}}_{i},y_{i}\right)}^{\left(t-1\right)}+y_{i}f_{t}(x_{i})+\frac{1}{2}h_{i}f_{i}^{2}(x_{i})]+\upsilon (f_{i}\right)$$

In Equation (4), $g_{i}=\partial_{y(t-1)}L({\ddot{y}}_{i},{\ddot{y}}^{t-1})$ represents the first-order gradient of the LF. $h_{i}=\partial_{y(t-1)}^{2}L({\ddot{y}}_{i},y^{\left(t-1\right)})$ represents the second-order gradient of the LF. The model weights are calculated as shown in Equation (5).

(5) $$w_{j}^{*}=-\frac{\sum_{i\in I_{j}}g_{i}}{\sum_{i\in I_{j}}h_{i}+\varphi }$$

In Equation (5), $I_{j}$ represents the sample set corresponding to leaf node j. $w_{j}^{*}$ represents the weight value. The model split gain calculation process is shown in Equation (6) [20,21].

(6) $$Gain=\frac{1}{2}[\frac{{\left(\sum_{i\in I_{L}}g_{i}\right)}^{2}}{\sum_{i\in I_{L}}h_{i}+\varphi }+\frac{{\left(\sum_{i\in I_{R}}g_{i}\right)}^{2}}{\sum_{i\in I_{R}}h_{i}+\varphi }-\frac{{\left(\sum_{i\in I}g_{i}\right)}^{2}}{\sum_{i\in I}h_{i}+\varphi }]-\lambda$$

**Figure 2:**
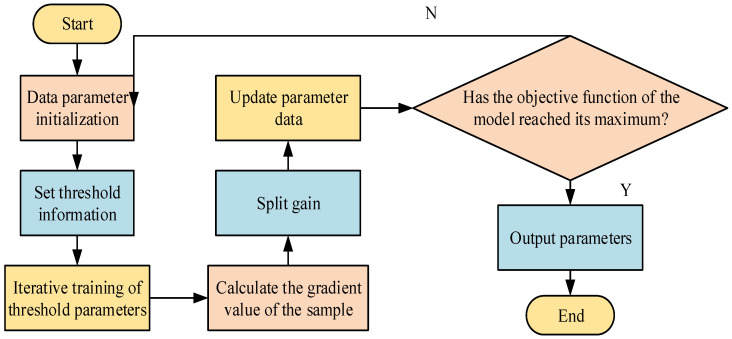
Algorithm running process.

In Equation (6), $I_{L}$ and $I_{R}$ display the sample sets of the left and right child nodes after splitting. The splitting condition of the model leaf must satisfy Gain>0 and the minimum number of LN samples. The algorithm’s operation process is shown in Figure 2.

In Figure 2, during data processing, the algorithm first initializes the data parameters and sets the initial threshold information. Then, the threshold parameters are iteratively trained. First, the sample gradient values and quadratic Taylor series expansions of each parameter data in the algorithm are calculated. Then, the split gain of the greedy selection split maximization model is maximized through the algorithm input. Lastly, the algorithm’s parameters are modified. The current algorithm objective function is then evaluated to determine whether it has reached its maximum value. If it has reached its maximum value, the parameter values of the current algorithm are output. If it has not reached its maximum value, the parameters are reinitialized, and the cycle is repeated. Based on the evaluation of the perceptual space of medical clothing, a clothing selection model is designed. Equation (7) is used to analyze the preferences of different groups of people in clothing selection [22,23,24].

(7) $$B_{Pi,Tj}=B_{o(Pi)}*w_{Pi,Tj}$$

**Figure 3:**
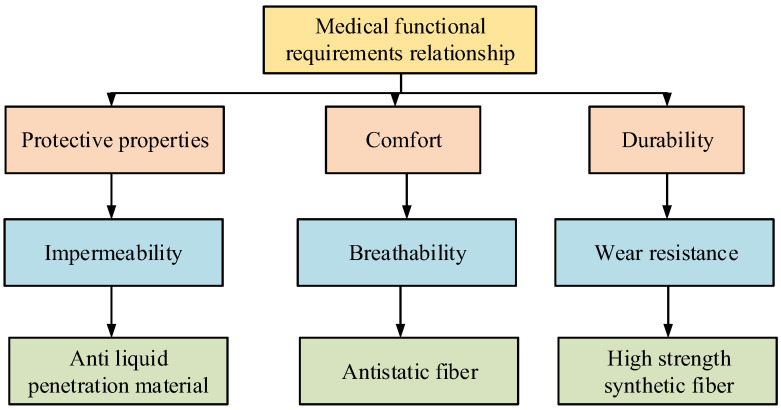
Corresponding relationship between clothing material selection and tendency.

In Equation (7), $B_{Pi,Tj}$ represents the degree of preference for clothing type i in the jth piece of clothing. $w_{Pi,Tj}$ indicates the degree of tendency of the $P_{i}$ personality corresponding to the $B_{Pi,Tj}=B_{o(Pi)}*w_{Pi,Tj}$ personality toward material Ti. $B_{o(Pi)}$ represents the initial degree of preference associated with the user’s personality type. Based on the degree of preference, the selection of medical clothing materials and the corresponding preferences are classified, as shown in Figure 3.

**Figure 4:**
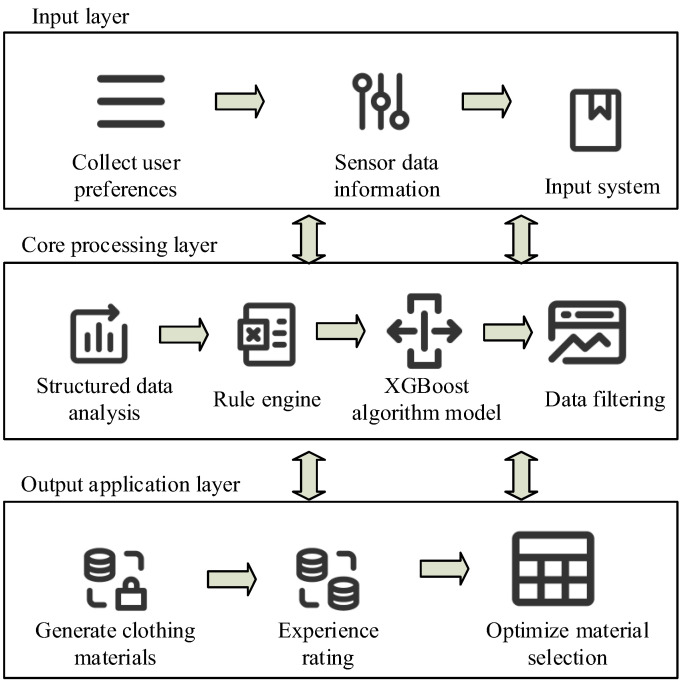
Personalized clothing material data selection perception model (Module source in the figure: https://iconpark.oceanengine.com/home, accessed on 8 January 2026).

In Figure 3, clothing selection within the intention space divides the corresponding relationships into four relationship layers. The first layer of relationships is the medical functional requirement relationship, which is further divided into three performance indicators: protection, comfort, and durability. Then, based on these performance indicators, they are categorized into waterproofing, breathability, and abrasion resistance. Finally, based on functional orientation, they are categorized into liquid-resistant materials, anti-static fibers, and high-strength synthetic fibers [25,26]. Based on clothing material selection, a personalized clothing material data selection perception model is constructed, as shown in Figure 4.

Figure 4 shows the clothing material selection model, which integrates medical scenario requirements, user physiological data, and environmental parameters. It combines these inputs with a material performance database and an intelligent matching engine to automate the process of making personalized recommendations based on data input. The system comprises three core modules: the data input layer (IL), the core processing layer, and the output application layer. The data IL first collects user preferences and sensor data information, which is then input into the system. The core processing layer performs structured data analysis using a rule engine and the XGBoost algorithm to process and filter the data. The output application layer generates clothing material solutions and scores them based on actual material selection experiences. Then, it reoptimizes material selection based on the scoring results. The proposed framework outputs a comprehensive applicability score for each candidate material rather than only a single final recommendation. Specifically, the XGBoost model integrates user-perception features, physical material parameters, and environmental coupling indicators to estimate the suitability of each material for the target application scenario. Candidate materials are then ranked according to their predicted scores to generate a Top-N recommendation list. In addition, feature-importance analysis is used to interpret the contribution of key variables, such as ionic-gel concentration, signal-to-noise ratio, skin–electrode impedance, and sweat resistance.

### 2.2. Design of an Environmental-Coupled Clothing Monitoring Board Based on Flexible Electronic Wearable Materials

**Figure 5:**
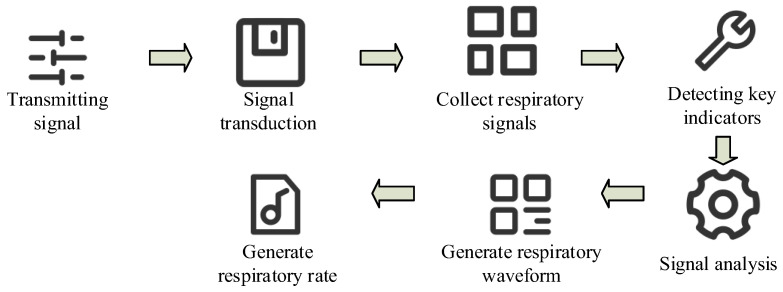
Medical respiratory monitoring process (Module source in the figure: https://iconpark.oceanengine.com/home, accessed on 8 January 2026).

A material selection model is used to recommend medical material fabrics so that clothing materials can adapt to the needs of different users. To make clothing suitable for medical monitoring environments, medical sensor devices are designed to achieve coupled monitoring within medical environments. Figure 5 shows the medical respiratory monitoring process.

In Figure 5, during respiratory monitoring, signals are transmitted via a high-frequency signal source and then conducted through wearable fabric electrodes. The fabric electrodes are used to collect respiratory signals and can detect key indicators such as respiratory rate, respiratory waveform, and impedance changes. Among these, the discrete Fourier transform (DFT) is used for signal analysis, and DC represents changes in the direct current component. The fabric electrode generates impedance upon receiving the electrode signals, forming respiratory waveforms through impedance changes. Ultimately, these signals are used to generate the respiratory rate to monitor respiration.

**Figure 6:**
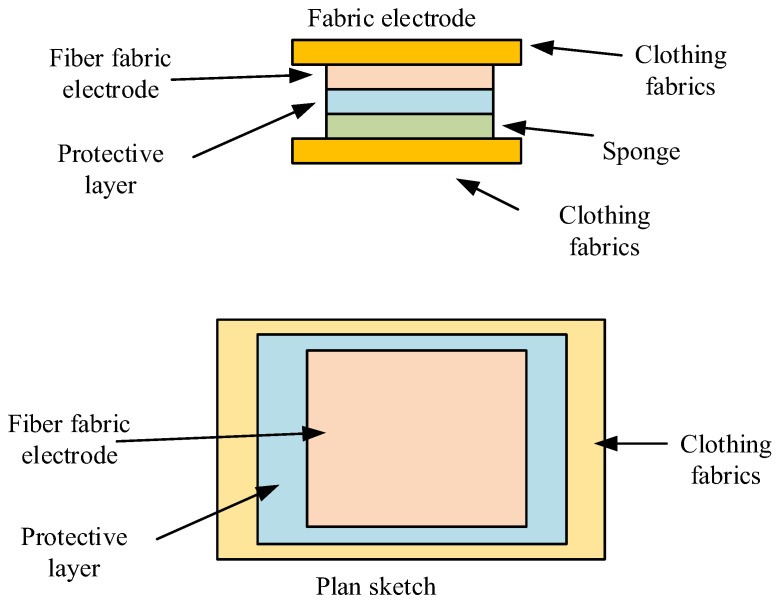
Electrode structure.

During gas exchange, the thoracic cavity expands and contracts rhythmically with inhalation and exhalation. During this process, the thoracic cavity’s volume and radius of curvature change [27,28]. Detecting these changes in curvature allows for the accurate acquisition of gas exchange frequency data [29,30]. This study investigates the use of a non-contact gas exchange monitoring device with a fork-shaped configuration, combining the unique design of high-precision gas detection elements with the outstanding performance of non-contact capacitive sensing technology for mechanical signal detection. The monitoring device employs a multilayer structure for data sensing, which includes a substrate, electrodes, encapsulation, and a gel layer. The electrode layer is protected by an outer protective layer. The substrate layer serves as the carrier platform for the entire device, providing a mounting foundation for the conductive functional layers. The gel layer contains positive and negative charge transport channels, enabling the input and output of electrical signals. After achieving electrode signal conduction, the electrode sensors are integrated into the clothing, serving as the sensing foundation for analysis. For environment-coupled medical clothing, electrode sensor devices are placed at the four corners of the clothing to monitor the wearer’s ECG, respiration, body temperature, etc. The sensors are positioned in the chest and armpit areas of the clothing, and physiological data is collected through the sensors when the user breathes [31]. The electrode sensors are integrated into the clothing through an integrated design. Figure 6 shows the motor structure.

**Figure 7:**
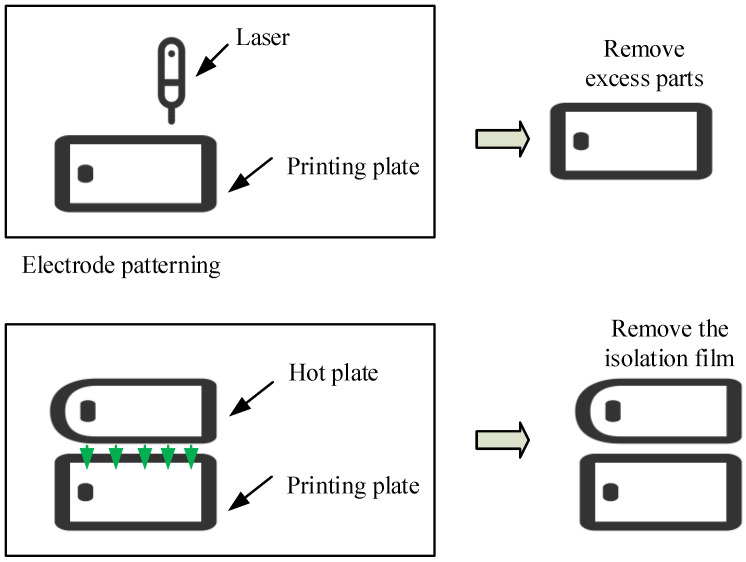
Preparation process of fabric electrode (Module source in the figure: https://iconpark.oceanengine.com/home, accessed on 8 January 2026).

In Figure 6, the electrode is divided into a four-layer structure, consisting of a fiber fabric electrode, a protective layer, a sponge layer, and a clothing fabric layer. The sensor’s nano-silver fiber fabric electrode layer directly contacts the skin, while the intermediate layer uses a sponge material with cushioning properties. The outermost layer is made of conventional clothing fabric to ensure comfort during wear. The sensor maintains close contact between the electrodes and the skin even during vigorous physical activity due to the elastic cushioning effect of the sponge layer, ensuring continuous and stable collection of electrocardiogram signals. The protective layer significantly extends the service life of the electrodes. To achieve the integration of electrodes with the fabric, a composite adhesive layer is used to prepare the fabric electrode. Figure 7 illustrates the fabrication process of the fabric electrode.

In Figure 7, during the preparation of the fabric, laser etching is first used to create the desired circuit pattern on the material surface. This is followed by a thermal transfer stage, where the etched pattern is heated at 170 °C for 3 min using a hot plate and transferred onto the target fabric substrate. Subsequently, post-processing is performed. First, any excess material that has not been transferred is removed. The material is then subjected to thermal pressing and curing again under the same temperature and time conditions. Finally, the polyethylene terephthalate (PET) release film is removed, and the entire preparation process is completed after cooling to room temperature. The entire process involves two precisely controlled thermal pressing steps, each maintained at 170 °C for 3 min.

### 2.3. Experimental Evaluation of Material Performance

To compare the comprehensive performance of candidate materials, several standardized tests were conducted. The signal-to-noise ratio was measured using an electrochemical workstation. Skin–electrode impedance was measured using a two-electrode method. Sweat resistance was evaluated by immersion in artificial sweat under 90% RH-equivalent conditions. Stretchability was assessed using a universal testing machine coupled with a resistance meter at 100% strain. Breathability was measured using an air permeability meter. Wear comfort was evaluated by 20 participants using a 5-point Likert scale, where 1 indicates very poor comfort and 5 indicates excellent comfort. The evaluation included softness, breathability, skin conformity, and overall wearing comfort. The final wear-comfort score for each material was calculated as the mean score across all participants and all evaluation dimensions. Unless otherwise stated, all tests were repeated three times, and the average values were reported. Representative commercial instruments suitable for these measurements include the Gamry Interface 1010E electrochemical workstation (Gamry Instruments, Warminster, PA, USA), the Hioki IM3536 LCR meter (Hioki E.E. Corporation, Ueda, Nagano, Japan), the Instron 5944 universal testing machine (Instron, Norwood, MA, USA), and the SDL Atlas AirPerm M021A air permeability tester (SDL Atlas, Rock Hill, SC, USA).

## 3. Results

### 3.1. Analysis of the Effects of Clothing Material Selection Model 

**Figure 8:**
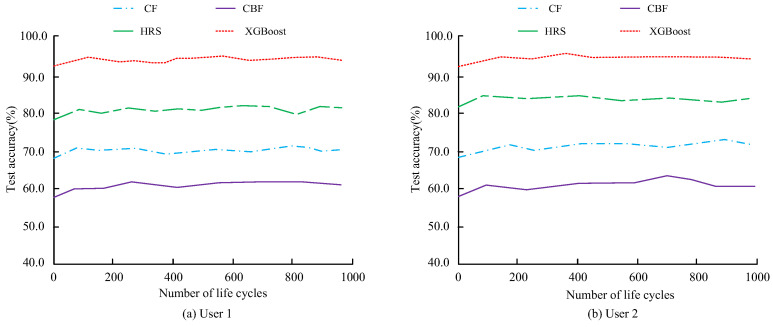
Comparison of testing accuracy among different participants.

For model training, a grid-search strategy combined with 5-fold cross-validation was used to optimize the XGBoost hyperparameters. The learning rate was searched within 0.01–0.3, the maximum tree depth within 3–10, the minimum child weight within 1–10, and the feature-sampling ratio within 0.5–1.0. Early stopping was applied when the validation performance did not improve for 50 consecutive rounds. The input dataset was constructed through multi-source fusion, including user-perception data obtained from structured questionnaires and converted to a 5-point scale, physical material parameters obtained from laboratory measurements and manufacturer datasheets, and environmental coupling performance indicators obtained from climate-chamber and physiological-monitoring experiments. After data cleaning and normalization, the dataset was divided into training and testing subsets at a ratio of 8:2. The final dataset contained 240 samples from five candidate material categories, including 192 training samples and 48 testing samples. Each sample corresponded to one material–condition evaluation instance formed by combining user-perception features, measured material parameters, and environmental coupling indicators. The final optimized parameter settings were as follows: learning_rate = 0.08, max_depth = 6, min_child_weight = 3, colsample_bytree = 0.8, and n_estimators = 300. This parameter combination achieved the best validation performance under the grid-search and 5-fold cross-validation setting. The comparative analysis of the selection accuracy of the CF algorithm, CBF algorithm, and HRS algorithm is shown in Figure 8.

In Figure 8a, the XGBoost model achieves the highest accuracy of 93.5% in the accuracy test of Subject 1. The detection accuracy of the CBF algorithm is relatively low, with a maximum value of only 62.1%, which is 31.4% lower than that of the XGBoost model. This shows that the XGBoost model has a good effect on clothing material recommendation, which may be due to the introduction of intention space to the model. In Figure 8b, the XGBoost model achieves the highest recommendation accuracy of 94.5% in the clothing recommendation test for Subject 2. The highest recommendation accuracy of the CBF algorithm is only 63.4%, which is 31.1% lower than that of the XGBoost model. The results indicate that, among different recommendation methods, the XGBoost model has higher recommendation accuracy and better recommendation performance. An auxiliary comparison of recommendation-related system performance across different models is provided in Table 1. User satisfaction is evaluated on a 5-point scale, with scores closer to 5 indicating better results.

**Figure 9:**
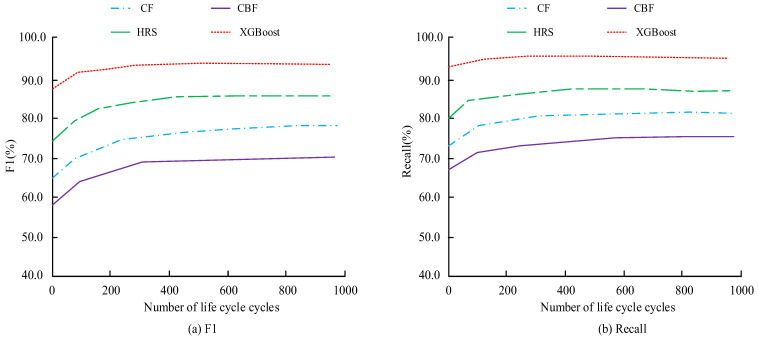
Comparison of model recall and F1 value.

Table 1 provides an auxiliary comparison of recommendation-related system performance across different models. To avoid over-reliance on platform-style metrics, the core model evaluation in this study is based on recommendation accuracy, recall, and F1 score, as further shown in Figure 9. Figure 9 compares the recall and F1 scores of different models under the same evaluation settings.

In Figure 9a, the F1 of different models increases with the number of iterations and tends to a relatively stable state after reaching a certain value. Among different models, the XGBoost model has the highest F1, reaching 92.1%. Meanwhile, the F1 of the CBF model is lower, with a maximum value of only 66.4%, which is 25.7% lower than that of the XGBoost model. This may explain why the XGBoost model has higher recommendation accuracy. In Figure 9b, in the recall analysis of different models, the recall of different models also increases with the number of iterations and tends toward a relatively stable state after reaching a certain value. The recall rate of the XGBoost model is the highest among several models, reaching 93.8%. The recall rate of the CBF model is the lowest among several models, with a maximum value of only 72.7%, which is 21.1% lower than that of the XGBoost model. Overall, across the different model performance tests, the XGBoost model exhibits superior performance in clothing material recommendation for users.

### 3.2. Multi-Performance Testing Results of Flexible Electronic Wearable Materials Coupled with Clothing Environment

This research selects AgNWs recommended by the model as the material for monitoring electrode plates in flexible electronic wearable devices. AgNWs exhibit ultra-high conductivity, excellent mechanical flexibility, good transparency, and lightweight characteristics. Meanwhile, the material can maintain stable electrical properties during deformation, significantly improving the quality of signal acquisition under dynamic motion. Moreover, its low skin-contact impedance and high signal-to-noise ratio enable it to accurately capture weak physiological signals. In the above content, the clothing material of subject 1 is subjected to a multi-performance analysis of clothing electrode materials. The coupling performance changes of the clothing materials under different environmental conditions are analyzed and tested. To test the performance of sensor electrodes in clothing materials, the influence of PVDF/[EMIM] [TFSI] ion-gel concentration on bending sensor sensitivity during preparation is studied. The performance changes of electrodes with different spacing are analyzed. Electrode sensing performance is tested separately at concentrations of 1:0.5, 1:1, and 1:2, as shown in Table 2. The sensing properties of the ionic gels with different ratios in Table 2 are measured using an LCR bridge tester after preparing the samples using the solution blending method. Initial capacitance and capacitance change are measured when the bending radius is 3 cm. Capacitance measurements are performed using an LCR bridge tester under ambient laboratory conditions (25 ± 1 °C, 50 ± 5% RH). Each condition is tested three times, and the average value is reported. Sensor sensitivity is evaluated based on the capacitance variation at a bending radius of 3 cm.

Table 2 shows that ion-gel concentration had a pronounced effect on sensor capacitance behavior. At a 1:0.5 ratio, the sensor exhibited limited capacitance variation, indicating insufficient sensitivity. At a 1:1 ratio, the electrode showed the most favorable balance between low initial capacitance and large bending-induced capacitance variation, and it was therefore selected as the optimal formulation. Although capacitance values further increase at the 1:1.5 and 1:2 ratios, the excessively high initial capacitance at higher concentrations reduced effective sensitivity and was unfavorable for stable practical sensing. These results indicate that ion-gel concentration should be optimized jointly with the electrode material when evaluating wearable sensing performance.

**Figure 10:**
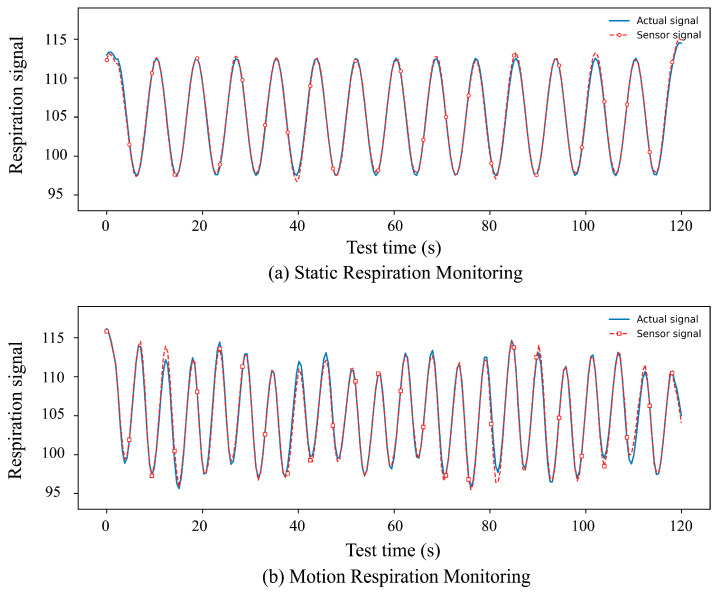
Respiratory monitoring results under different conditions: (**a**) static respiration monitoring; (**b**) respiration monitoring during motion.

To test changes in clothing detection and coupling performance in motion and stationary states, the breathing conditions of the same subject in both states were analyzed. Respiratory signals were continuously sampled at 50 Hz for both static and motion states. Each recording lasted 120 s. The signals shown in Figure 10 correspond to the respiratory waveforms directly used for comparison between the reference respiration signal and the sensor-measured signal. Physiological signals were acquired using a non-contact capacitive sensing system based on multilayer composite fabric electrodes, consisting of a nano-silver-fiber fabric electrode layer, a protective layer, a sponge elastic layer, and a clothing-fabric layer.

**Figure 11:**
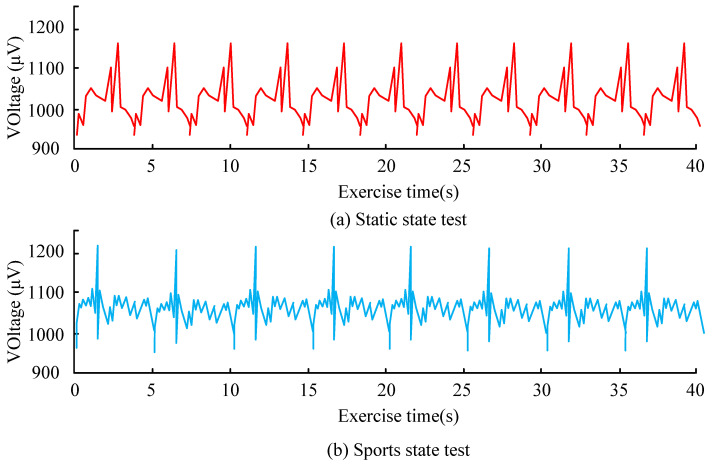
ECG monitoring results under static and motion conditions.

Figure 10 presents the respiratory monitoring results under static and motion conditions. In the static state (Figure 10a), the sensor-measured signal closely follows the reference respiration waveform, showing stable periodic characteristics and high consistency in amplitude and phase. In the motion state (Figure 10b), the measured signal exhibits slightly increased fluctuations due to body movement and mechanical disturbance; however, the overall waveform remains well aligned with the reference signal. The deviation between the measured and reference signals remains within approximately 3%, demonstrating that the proposed textile sensor can achieve reliable respiratory monitoring under both static and dynamic conditions. To further analyze the environmental coupling performance of the clothing materials, monitored ECG signals were examined. ECG signals was acquired at 250 Hz and processed using a 0.5–40 Hz band-pass filter. Figure 11 shows the ECG monitoring performance under static and motion conditions.

Figure 11 shows the ECG monitoring performance under static and motion conditions. In the static state, the signal exhibited the highest stability, with an R-wave amplitude of 1166 μV, a sensitivity of 97.9%, and a signal-to-noise ratio of 4.2 dB. Under motion, the ECG waveform remained identifiable, although the signal fluctuated more noticeably because of body movement and frictional interference, and the signal-to-noise ratio decreased to 3.4 dB. The R-wave amplitude increased slightly to 1185 μV during motion, while detection sensitivity decreased because of motion artifacts. Overall, these results suggest that the selected textile material and multilayer electrode structure can still support stable physiological monitoring in dynamic environments, although motion introduces additional noise and reduces signal quality to some extent. After analyzing the changes in the coupling of clothing materials and using the clothing selection model, the actual operational effects are shown in Table 3.

Table 3 shows that the XGBoost selection model improved signal quality and signal-to-noise ratio by 68% after using the clothing material selection model. Contact impedance is reduced by 68%, significantly improving the accuracy of physiological signal acquisition. In terms of the mechanical performance of the model, using XGBoost to select materials reduced changes in tensile resistance by 73% and improved skin adhesion by 24%. This enables the material to ensure stable monitoring during intense exercise. In terms of environmental adaptability, sweat resistance performance improved by 70%, and temperature-induced signal drift reduced by 73%. This shows that the use of the XGBoost selection model solves the failure problem of traditional materials in complex environments. Compared to using non-XGBoost to select models, the comfort rating of users increased by 44%, and 24-h sensitivity attenuation decreased by 75%. This shows that after using the material selection model, there is a significant improvement in both the usage effect and environmental coupling effect of the entire clothing material. Wear comfort was quantified based on participant ratings, as described in Section 2.3, and then normalized together with objective performance indicators before being input into the XGBoost model. Rather than assigning a fixed manual weight to comfort alone, the model learned the relative importance of comfort-related and sensing-related features across different application scenarios. In this way, the final recommendation score reflected both user-rated comfort and objective environmental coupling performance. All standardized subjective and objective indicators were input into the XGBoost model to calculate the comprehensive applicability score for each candidate material. Based on this integrated evaluation, AgNWs achieved the highest overall score and were selected for further validation. The performance of AgNWs was then compared with other representative flexible wearable materials, as summarized in Table 4. The comprehensive performance of different candidate materials was evaluated as follows: signal-to-noise ratio was measured using an electrochemical workstation; skin–electrode impedance was measured using a two-electrode method; sweat resistance was evaluated by immersion in artificial sweat; stretchability was assessed using a universal testing machine coupled with a resistance meter; and breathability was measured using an air permeability meter. Wear comfort was quantified according to the protocol described in Section 2.3.

As summarized in Table 4, AgNWs exhibited the best overall environmental coupling performance among the candidate materials. The signal-to-noise ratio reached 5.2 dB, which was markedly higher than that of PEDOT:PSS (3.2 dB). AgNWs also showed the lowest skin–electrode impedance (3 kΩ/cm^2^), indicating superior interfacial electrical coupling. In the sweat-resistance test, AgNWs exhibited only an 8% impedance variation, suggesting stronger stability under humid conditions compared to the other materials. In addition, AgNWs achieved the highest conformability (95%) and wear-comfort score (4.8) while maintaining acceptable mechanical stability. These results indicate that AgNWs provide a more balanced combination of sensing performance, environmental adaptability, and wearability than the other tested materials. The comparison of indicator results across different models is shown in Table 5.

Table 5 shows that the full XGBoost model achieved the best overall performance, with an accuracy of 94.5%, a recall of 93.8%, and an F1 score of 92.1%. Removing user-intention features caused the largest performance drop, indicating that subjective preference information plays an important role in material recommendation. Removing environmental coupling features also reduced model performance, confirming the necessity of integrating real-world material–environment interaction indicators. These findings support the effectiveness of multidimensional feature fusion in the proposed framework.

## 4. Conclusions

This study proposed a data-driven material selection framework for flexible wearable sensors under environmental coupling conditions. By integrating user perception, material physical parameters, and environmental coupling indicators, the XGBoost-based model achieved superior recommendation performance compared with conventional methods. The recommended AgNW material also demonstrated strong sensing performance, low skin-contact impedance, and good environmental adaptability in physiological monitoring experiments. Nevertheless, this study has several limitations. First, the current model was validated on a limited range of candidate materials and application scenarios. Second, long-term biocompatibility and stability under more complex real-world conditions require further investigation. Future work will expand the material database, refine the scoring mechanism, and validate the framework in broader wearable-monitoring applications.

## Figures and Tables

**Table 1:** Performance comparison of different models.

Evaluation Metric	CF	CBF	HRS	XGBoost
Click-through rate (CTR)	12%	10%	15%	18%
Conversion rate	5%	3%	7%	9%
User satisfaction	4.1	3.8	4.3	4.6
Dwell time	45 s	35 s	55 s	65 s
User retention rate	25%	18%	30%	35%
Response time	120 ms	50 ms	80 ms	30 ms
Throughput (QPS)	500	1200	800	2000
Cold start coverage	20%	60%	50%	75%

**Table 2:** Comparison of electrode sensing performance.

PVDF/[EMIM] [TFSI] Ionic Concentration Ratio	Initial Capacitance (pF)	Capacitance at 3 cm Bending Radius (nF)	Sensitivity Trend Description
1:0.5	50	4	Low concentration, small capacitance change
1:1	10	66	Optimal sensitivity, selected as best ratio
1:1.5	200	104	Increased concentration leads to significantly higher capacitance
1:2	300	123	High concentration causes excessive initial capacitance, reducing sensitivity

**Table 3:** Analysis of the actual operation effect of the model.

Performance Metric	Non-XGBoost	XGBoost Model
Signal-to-noise ratio (SNR)	2.5 dB	4.2 dB
Signal stability	±15%	±5%
Skin–electrode impedance	25 kΩ/cm^2^	8 kΩ/cm^2^
Stretch tolerance	+30% at 100% strain	+8% at 100% strain
Skin conformability	75%	93%
Sweat resistance	+50% at 90% humidity	+15% at 90% humidity
Temperature stability (10–40 °C)	±3 mV	±0.8 mV
Motion artifact suppression	30% noise ratio	12% noise ratio
Wear comfort	3.2	4.6
Long-term durability (24-h sensitivity)	20% degradation	5% degradation

**Table 4:** Comparison of properties of different materials.

Performance Metric	Graphene	PEDOT:PSS	CNTs	Liquid Metal	AgNWs
SNR (dB)	3.8	3.2	4	4.5	5.2
Skin–electrode Impedance (kΩ/cm^2^)	15	20	12	5	3
Sweat resistance (impedance variation, %)	25%	40%	18%	10%	8%
Temperature stability (signal drift, mV)	±1.5	±2.0	±1.2	±0.9	±0.5
Motion artifact suppression (noise ratio, %)	15%	22%	13%	8%	5%
Stretchability (resistance change at 100% strain, %)	20%	10%	25%	50%	5%
Conformability (%)	85%	80%	88%	75%	95%
Breathability (air permeability, mm/s)	600	450	550	300	700
Wear comfort *	4.2	3.9	4.3	3.5	4.8
Durability (bending cycles)	800	600	700	400	>1200

* Wear comfort was rated on a 5-point Likert scale (1 = very poor, 5 = excellent). Values are reported as mean scores across 20 participants.

**Table 5:** Comparison of evaluation indicators for different models.

Model Configuration	Recommendation Accuracy (%)	Recall (%)	F1 Value (%)
Full XGBoost model	94.5	93.8	92.1
Remove user intention features	86.2	85.1	83.7
Remove environmental coupling features	89.3	88.5	87
Material parameters only	78.6	77.2	75.9
LightGBM	93.8	92.9	91.5
CatBoost	94.1	93.2	91.8
DeepFM	94.3	93.5	92

All model comparisons were performed on the same dataset split and under the same evaluation protocol.

## Data Availability

The dataset is available on request from the authors.
